# GDF-15: Can It Be Used as a Biomarker in Acute Cerebrovascular Incidents?

**DOI:** 10.3390/jpm16060300

**Published:** 2026-06-01

**Authors:** Areti Kourti, Eirini Keskilidou, Alexandra Skoura, Paraskevi Karalazou, Katerina Thisiadou, Kali Makedou

**Affiliations:** Laboratory of Biochemistry, AHEPA University Hospital, School of Medicine, Faculty of Health Sciences, Aristotle University of Thessaloniki, 54124 Thessaloniki, Greece; aretikourti@auth.gr (A.K.); eirinikeski@hotmail.gr (E.K.); alexskoyra@gmail.com (A.S.); vivikarala@gmail.com (P.K.); thisiadou@yahoo.gr (K.T.)

**Keywords:** GDF-15, suPAR, CRP, acute cerebrovascular events, stroke, inflammation

## Abstract

**Background/Objectives:** Growth differentiation factor-15 (GDF-15) is a protein that belongs to the transforming growth factor beta superfamily and has been found elevated in cases of organ injury such as liver, kidney, heart, and lung, as well as cardiovascular diseases and cancer. Soluble urokinase plasminogen activator receptor (suPAR) is a protein which is expressed mainly on immune cells and endothelial and smooth muscle cells, and is a marker of severity and intensity of inflammation in acute and chronic diseases. The aim of the present study was to compare GDF-15 serum levels between patients with acute cerebrovascular incidents and healthy controls and to investigate the possible correlation of GDF-15 serum levels and inflammatory markers, such as serum C-reactive protein (CRP) and plasma suPAR, in the above-mentioned groups. **Methods:** This is a retrospective study. Thirty-one patients were included in the study, with a mean age ± SD of 67 ± 13 years, compared to 18 age-matched healthy controls. **Results:** In the patient group a statistically significant positive correlation of serum levels of GDF15 values with suPAR and CRP emerged (rs = 0.516, *p* = 0.003) and (rs = 0.409, *p* = 0.022), respectively, and no significant correlation was found in the group of controls (rs = 0.271, *p* = 0.277) and (rs = 0.423, *p* = 0.080), respectively. **Conclusions:** These findings support the role of inflammation as a key underlying mechanism in acute cerebrovascular injury and suggest that GDF-15 may serve as a valuable adjunct biomarker for assessing disease severity and inflammatory burden.

## 1. Introduction

Growth differentiation factor-15 (GDF-15) is a protein that belongs to the transforming growth factor beta (TGF-β) superfamily. It is a 308aa peptide consisting of a signal peptide, a propeptide, and a mature peptide. It contains a 9-cycteine region which stabilizes the GDF monomer. In the endoplasmic reticulum (ER), the 6th cysteine forms a disulfide bond with the 6th cysteine from another pro-GDF-15 monomer to form a pro-GDF-15 homodimer. After exiting ER, proteolytic cleavage takes place and mature homodimer GDF-15 is released [1].

Normally, it is expressed at relatively low levels in most tissues and in many tissues under stressful conditions. It has been found elevated in cases of organ injury, like liver, kidney, heart, and lung [1,2]. Only recently has its role been partly described, when different research groups identified its receptor, an alpha-like proto-oncogene tyrosine kinase receptor Ret (GFRAL-RET), in the hindbrain region acting in the inhibition of food intake [3]. These results indicate that GDF-15 exerts actions probably in various other regions of the brain.

GDF-15 is now increasingly considered a new biomarker related to an elevated risk of cardiovascular diseases and cancer. It is expressed at low levels in most tissues, whereas in the placenta, the prostate, and some of the abdominal viscera high levels have been found under physiological conditions [4]. However, in tissue injury GDF-15 is impressively increased. For example, in brain injury experimental models, there is an increase in GDF-15 expression in many areas and cells of the nervous system, such the neurons, apart from the choroid plexus of adult rat brain that is normally observed [5]. Moreover, the fact that the expression of GDF-15 is elevated in macrophages can explain the increase in GDF-15 during stress stimuli on different cells, such as brain cells during brain injury or adipose tissue cells during high-fat diet [6].

Soluble urokinase plasminogen activator receptor (suPAR) is the soluble form of uPAR, a protein expressed mainly on immune cells and endothelial and smooth muscle cells. It can be detected in blood, cerebrovascular fluid, saliva, or urine and plays a central role in plasminogen activation and fibrinolysis and participates in inflammatory processes, cell proliferation, migration, and adhesion, as well as angiogenesis [7]. In reference to the structure of suPAR and uPAR, they share similar structure. They have three domains (D1–D3) with a connecting region between D1 and D2 that includes cleavage sites for proteases’ action (chymotrypsin, elastase, matrix metalloproteinases, cathepsin G, plasmin, uPA). They also have a glycosyl phosphatidylinositol (GPI) anchor that after cleavage can result in three suPAR isoforms, as follows: suPAR I-III, suPAR I, suPAR II-III. SuPAR presents variations due to post-translational glycosylation [8,9].

During inflammation, uPAR is cleaved from immune cell surface and suPAR is created. Therefore, there is a strong relation between suPAR levels and immune system activation. UPAR is currently being used as a marker of severity and intensity of inflammation in acute and chronic diseases [10,11,12]. Most pro-inflammatory cytokines are of limited clinical value, due to short half-life, circadian fluctuations, and susceptibility to variations according to dietary intake and physical activity. On the other hand, suPAR is a more stable protein, with minimal circadian fluctuations. It appears that it is positively correlated with traditional markers of inflammation, such was C-reactive protein (CRP), IL-6, and TNF-a, and women present slightly higher suPAR levels than men. Nevertheless, CRP and suPAR seem to reflect different aspects of inflammation; CRP is a marker of acute infection and metabolic inflammation and is strongly correlated with BMI, whereas suPAR has been associated with cardiometabolic risk factors and weakly associated with BMI [8].

SuPAR also increases with age and presents higher in serum than in plasma [13]. Recent data indicate that apart from acute inflammation and sepsis, suPAR may be a useful biomarker for systematic chronic inflammation (SCI), characterizing health issues like cardiovascular diseases, diabetes mellitus type 2, cancer, and neurodegenerative and mental health disorders [14]. So far, SCI levels are measured by the combination of biomarkers of inflammation and infection and lack standard biomarkers for the assessment of its severity.

Various studies have shown that suPAR is associated with risk factors and outcomes of systemic chronic inflammation independently of common inflammation markers [8]. suPAR and CRP share common characteristics and differ in others. Some common features are as follows: they are both released in inflammatory conditions and infections, they are produced by the liver and other tissues and organs after the influence of pro-inflammatory cytokines, and they are non-specifically associated with multiple diseases but are both linked to a higher risk of adverse outcomes. Nevertheless, these two biomarkers differ in their susceptibility to acute and chronic stress factors, time of release, response kinetics like amplitude and stability of release, and the type of clinical conditions that they are strongly related to. In particular, CRP is an excellent biomarker of bacterial infection rising to up to 1000-fold within 24–72 h of a bacterial infection, whereas suPAR is an inferior diagnostic marker for the distinction between bacterial and non-bacterial infections or sepsis. Moreover, in contrast to CRP, suPAR seems to be associated with early-life risk factors and stressful experiences in childhood, and remains associated with disease mortality even when levels of CRP are low (<10 mg/L) [8].

The aim of the present retrospective study was to compare GDF-15 serum levels between patients with acute cerebrovascular incidents and healthy controls. Furthermore, the study aimed to investigate the possible correlation of GDF-15 serum levels and inflammatory markers, such as serum CRP and plasma suPAR, in the above-mentioned groups.

## 2. Materials and Methods

### 2.1. Characteristics of the Study Sample

This is a retrospective study including in total 31 patients with acute cerebrovascular events that presented in the ER, 16 men and 15 women with a mean age ± SD of 67 ± 13 years, as well as 18 healthy controls of similar age. Data of all patients were extracted from the archives of our hospital. Samples were collected in tubes without anticoagulant, for the isolation of serum, and in tubes with EDTA, as anticoagulant, for plasma isolation, and were processed for determinations within an hour, due to the emergency of the cases. All patients presented or were brought in the ER of our hospital.

### 2.2. Determinations

The concentration of serum GDF-15 was determined by electrochemiluminescence (ECLIA), using a sandwich principle, with the immunological analyzer e801 of the automated system COBAS 8000 (Roche Diagnostics, Mannheim, Germany). In specific, human serum was diluted 1:5 automatically and two kinds of antibodies were used, as follows: a biotinylated monoclonal anti-GDF-15 antibody (mouse) 1.5 µg/mL and a monoclonal anti-GDF-15 antibody (mouse) labeled with ruthenium complex 2.0 µg/mL. The measuring range of the assay is typically up to 20,000 pg/mL, with a lower detection limit of 400 pg/mL. Intra-assay precision ranges from 0.7% to 2.2%, and inter-assay precision is ≤4.6%.

Plasma suPAR levels were determined by particle-enhanced turbidimetric immunoassay (PETIA) (suPARnostic TurbiLatex Reagents, Virogates, Birkerod, Denmark) on the biochemical analyzer e702 of the automated system COBAS 8000 (Roche Diagnostics, Mannheim, Germany). The kit consists of two reagents which are loaded into the analyzer on an analyzer-dedicated reagent cassette. The analyzer automatically mixes the reagents that consist of latex-enhanced particles coated with anti-suPAR antibodies (mouse/rat), which agglutinate with suPAR present in the sample. The size of the antigen–antibody complex that is formed is estimated by spectrophotometric technology at 570–590 nm. The degree of the turbidity caused by agglutination is proportional to the concentration of suPAR levels in the sample.

CRP levels were quantified in serum samples with a latex-enhanced immunoturbidimetric method on DxC 700 AU automated analyzer (Beckman Coulter Ireland Inc., Clare, Ireland). The assay measures turbidity changes proportional to CRP concentration. Specific antibody-coated latex particles bind CRP in the sample, causing agglutination and turbidity, which is measured spectrophotometrically. The CRP reagent is linear from 5 to 300 mg/dL. The inter-assay precision ranges between 0.7 and 2.1%CV, and total precision is less than 5%CV.

### 2.3. Statistical Analysis

Statistical analysis was performed with the IBM SPSS v.25 software, and *p* < 0.05 was considered as the level of statistical significance. The Kolmogorov–Smirnov test was used for testing normality of distribution of values. The Mann–Whitney test was applied for non-parametrical between-group comparisons, and Spearman’s correlation test provided results for within-group correlations between values of parameters.

### 2.4. Ethical Aspects

The study was conducted in accordance with the Declaration of Helsinki, and approved by the Scientific Board of AHEPA University Hospital of Thessaloniki, Greece (code 673, approved on 23 December 2025). Informed consent was waived due to the retrospective nature of the study.

## 3. Results

**Figure 1:**
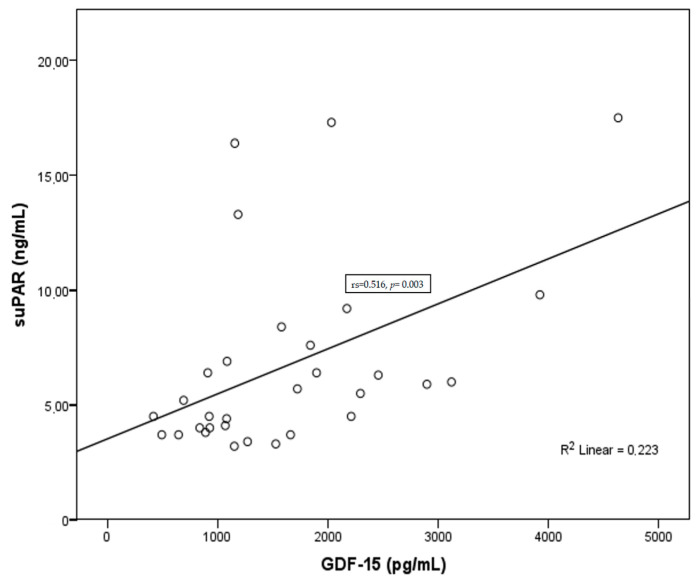
Scatter plot depicting significant positive correlation of values of GDF-15 (pg/mL) and suPAR (ng/mL) in the group of patients with acute cerebrovascular events.

**Figure 2:**
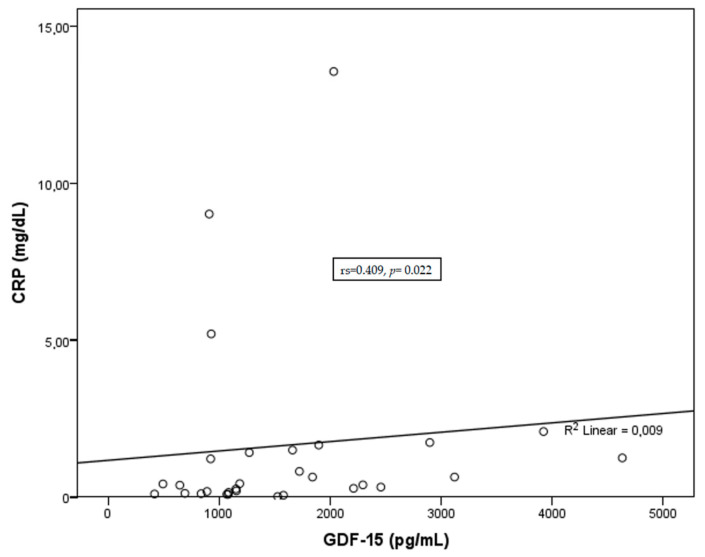
Scatter plot depicting significant positive correlation of values of GDF-15 (pg/mL) and CRP (mg/dL) in the group of patients with acute cerebrovascular events. CRP: C-reactive protein.

The results of the determinations of GDF-15, CRP, and suPAR levels in the two groups are shown in Table 1. All biomarkers of inflammation were found significantly more elevated in patients with acute cerebrovascular incidents than in the controls. In the patient group, a statistically significant positive correlation of serum levels of GDF-15 values with plasma suPAR (rs = 0.516, *p* = 0.003) and serum CRP levels (rs = 0.409, *p* = 0.022) emerged (Figure 1 and Figure 2). No such significant correlation was found in the group of controls (rs = 0.271, *p* = 0.277) and (rs = 0.423, *p* = 0.080), respectively, although correlation was positive.

## 4. Discussion

The present study investigated the potential role of GDF-15 as a novel biomarker in acute cerebrovascular events and demonstrated that serum GDF-15 levels are significantly elevated in patients compared with healthy controls. Moreover, the observed positive correlations between GDF-15 and established inflammatory biomarkers (CRP and suPAR) further support the concept that GDF-15 reflects inflammatory activation in the acute phase of cerebrovascular injury.

GDF-15 is a dimer with a molecular weight of 25 kD containing an inter-chain disulphide bond and significant structural differences from all the other members of the TGF superfamily [15]. It has been related to inflammation, hypoxia, and tissue injury and remodeling and is secreted as a result of different stress stimuli, positioning it as an integrative marker of cellular stress. NF-κΒ is the transcription factor complex that has been found to directly regulate GDF-15 expression along with other pro-inflammatory factors [15,16]. Hypoxia of altitude has been found to promote GDF-15 secretion and is related to increased levels by 50% after 24 h at [17].

GDF-15 has been investigated as a possible biomarker for many organ and system pathologies. Even slightly increased levels above normal have been linked to all-cause mortality [18]. High GDF-15 levels have been found in cancers like prostate, colorectal, and pancreatic and in malignant gliomas, and seems to play a role in cancer cachexia as well [3]. GDF-15 levels are also an independent factor predicting adverse outcomes of cardiovascular diseases, and they indicate high mortality in patients with ST-segment elevation acute coronary syndrome (STE-ACS), non-STE-ACS, and heart failure [19,20,21]. Moreover, elevated GDF-15 levels are indicative of increased risk of developing heart failure after an acute coronary event [22]. Additionally, GDF-15 is considered a useful biomarker for the prevention of bleeding in atrial fibrillation (AF) patients under anticoagulation treatment for the prevention of stroke and death. ABC bleeding risk score, considering GDF-15, high-sensitivity troponin, hemoglobin, and age, has been acknowledged as a valuable decision-supporting tool [23,24].

GDF-15 has been investigated by the research group of Gadd AD, et al. in conditions affecting the brain. Their research showed that GDF-15 may be used in scores, like EpiScore, as potential tools of risk stratification [25]. Increased levels of GDF-15 were found in patients after a stroke [26], which is in accordance with the findings of our study. Lee J. et al., summarizing recent advances in biomarkers of ischemic stroke, referred to GDF-15 as a marker depicting infarct size and predicting long-term outcomes [27]. The research group of Zhou Y et al. reviewed existing literature in reference to GDF-15 as a potential therapeutic target for brain diseases and found that inhibiting GDF-15 can suppress tumor progression. Another finding of the team of Guo D. was that GDF-15 can predict poor clinical outcomes in acute ischemic stroke [28,29]. Acute stroke is characterized by a complex pathophysiological cascade involving excitotoxicity, oxidative stress, endothelial dysfunction, microglial activation, and systemic immune response. Therefore, it seems that GDF-15 assayed in serum of patients with stroke could provide additional data for screening ischemic stroke patients that are at high risk for poor prognosis.

suPAR, as a protein detected in plasma, serum, and other biological fluids, is involved in many physiological processes, such as proliferation, migration, adhesion, angiogenesis, and inflammation [7]. Acute conditions and chronic diseases, such as kidney disease, cardiovascular disease, sepsis, and inflammatory conditions, have been associated with increased suPAR plasma levels. Nevertheless, suPAR has not been acknowledged as a disease-specific diagnostic marker yet, but it can predict morbidity and mortality of systemic chronic inflammation, as it correlates with established inflammatory biomarkers, and is elevated in cases of immune activation [29,30,31]. Recent data indicate that increased suPAR levels are associated with poor outcomes in cases of acute brain injury, stating a role as a potential prognostic biomarker across different types of brain injury [32], and this is in accordance with our study showing significantly more elevated plasma levels in patients than controls.

Moreover, several studies have shown a significant increase in GDF-15 levels in neurologic disorders. In specific, children with hemorrhagic shock or encephalopathy have been shown to present with four hundred times higher GDF-15 levels than controls [33]. These findings were also common in clinical conditions, like status-epilepticus and migraines in children [34,35].

The present study investigated the possibility of GDF-15 becoming a novel biomarker, in addition to suPAR and CRP, for acute vascular cerebral incidents. This differentiation factor seems to be significantly more increased in patients than controls and presented significant positive correlation to the above-mentioned classic markers of inflammation that were also determined. Our results are in accordance with those of Méloux et al. [36], who found that GDF-15 gene and pro-protein are expressed in the ischemic brain after a stroke, but in general not many data exist on this field of research. The significant correlation of GDF-15 to inflammatory biomarkers reveals a link between various inflammation pathways underlying acute cerebrovascular events. Although both CRP and suPAR are non-specific inflammatory biomarkers, the combination of the three analytes that were presently studied could both enlighten pathophysiology and help toward prediction of the progression of patients’ conditions.

From a translational standpoint, GDF-15 may offer several potential clinical applications that have to be further investigated. Its early elevation could aid in risk stratification at hospital admission, particularly when integrated into multimarker models incorporating inflammatory, cardiac, and coagulation parameters (CRP, suPAR, troponin, NT-proBNP), aiming at individualized prognostic assessment as well. Furthermore, given the established role of GDF-15 in bleeding stratification in cardiovascular disease and atrial fibrillation [37], future research should investigate whether elevated GDF-15 levels are associated with hemorrhagic transformation after thrombolysis or mechanical thrombectomy. Moreover, serial measurement of GDF-15 during hyperacute and subacute phases might provide insight into dynamic inflammatory and injury responses. In this frame, GDF-15 may be investigated as a possible biomarker of ongoing injury processes, when elevated, or of neurological recovery, when its levels decline. Lastly, in terms of secondary prevention, elevated post-stroke GDF-15 levels may identify patients at increased risk of recurrent cerebrovascular events, potentially guiding aggressive secondary prevention strategies, and this remains to be investigated in future studies. Whether GDF-15 acts as a protective mediator limiting excessive inflammation or as a marker of irreversible injury also remains to be clarified.

The present study has revealed several limitations that should be carefully considered when interpreting the findings. First, the relatively small sample size limits statistical power and restricts the generalizability of the results. As a single-center retrospective analysis, the study may be subject to selection bias and may not fully represent the broader population of patients with acute cerebrovascular events. Second, the retrospective design of the study precludes establishment of causal relationships and limits control over potential confounding variables. Important clinical parameters, such as stroke subtype (ischemic versus hemorrhagic), infarct volume, NIHSS score at admission, timing of blood sampling relative to symptom onset, reperfusion therapies, and functional outcome measures were not systematically incorporated into the analysis. Consequently, we were unable to determine whether GDF-15 independently predicts stroke severity, short-term complications, or long-term outcomes. As a third limitation of the study we could mention the fact that multiple comorbid conditions, such as heart failure, atrial fibrillation, renal dysfunction, malignancy, and advanced and systemic inflammatory diseases, were not adjusted for multivariate analyses and may have contributed to inter-individual variability in biomarker levels. Although inflammatory markers were elevated collectively in the patient group, residual confounding cannot be excluded. Fourth, only single time-point measurements were analyzed. Serial measurements would provide valuable information regarding the dynamic profile of GDF-15 during the hyperacute, acute, or subacute phase of stroke. Finally, although significant correlations were observed between GDF-15 and established inflammatory biomarkers (CRP and suPAR), correlation does not imply mechanistic interaction. The study does not elucidate whether GDF-15 is merely a marker of systemic stress or an active mediator in the pathophysiological cascade of cerebrovascular injury.

Despite these limitations, the present investigation provides preliminary evidence supporting the potential relevance of GDF-15 in acute cerebrovascular events and establishes a foundation for future large-scale, prospective, and mechanistically oriented studies.

## 5. Conclusions

In conclusion, the present study demonstrates that serum GDF-15 levels are significantly elevated in patients with acute cerebrovascular events and are positively correlated with established inflammatory biomarkers, including CRP and suPAR. These findings support the role of inflammation as a key underlying mechanism in acute cerebrovascular injury and suggest that GDF-15 may serve as a valuable adjunct biomarker for assessing disease severity and inflammatory burden.

From a clinical perspective, the strong association between GDF-15 and markers of immune activation highlights its potential utility in early risk stratification, identification of patients at higher risk of adverse outcomes, and refinement of prognostic evaluation of patients with acute cerebrovascular incidents. Incorporation of GDF-15 into multimarker panels alongside CRP and suPAR could enhance the accuracy of inflammatory burden assessment and contribute to more individualized patient management.

However, before clinical implementation can be recommended, larger prospective studies are required to validate its independent prognostic value, define standardized cut-off levels, assess its performance across stroke subtypes, and determine the utility of serial measurements in monitoring disease progression and therapeutic response. Overall, GDF-15 represents a promising biomarker candidate in acute cerebrovascular events, but further research is essential to clarify its precise clinical role and potential therapeutic implications.

## Figures and Tables

**Table 1:** Serum levels of GDF-15 and CRP, and plasma suPAR levels in patients with acute vascular cerebral episodes and healthy controls.

	GDF-15 (pg/mL)	CRP (mg/dL)	suPAR (ng/mL)
	Median (min–max)		
Patients, Ν = 31	1270 (416–4632)	0.42 (0.02–13.56)	5.5 (3.2–17.5)
Healthy controls, Ν = 18	616 (400–1324)	0.09 (0.02–2.71)	3.8 (2.2–7.5)
*p*	<0.001 °	0.001 °	0.004 °

°: statistically significant difference.

## Data Availability

The data are not publicly available due to data protections regulations.

## Abbreviations

The following abbreviations are used in this manuscript:

GDF-15	Growth differentiation factor-15
suPAR	Soluble urokinase plasminogen activator receptor
CRP	C-reactive protein

## References

[1] Liuizė Abramavičiūtė A., Mongirdienė A. (2024). TGF-β Isoforms and GDF-15 in the Development and Progression of Atherosclerosis. Int. J. Mol. Sci..

[2] McDowell K., Campbell R., Simpson J., Cunningham J.W., Desai A.S., Jhund P.S., Lefkowitz M.P., Rouleau J.L., Swedberg K., Zile M.R. (2023). Incremental prognostic value of biomarkers in PARADIGM-HF. Eur. J. Heart Fail..

[3] Lockhart S.M., Saudek V., O’Rahilly S. (2020). GDF15: A Hormone Conveying Somatic Distress to the Brain. Endocr. Rev..

[4] Castiglione V., Aimo A., Vergaro G., Saccaro L., Passino C., Emdin M. (2022). Biomarkers for the diagnosis and management of heart failure. Heart Fail. Rev..

[5] Schober A., Böttner M., Strelau J., Kinscherf R., Bonaterra G.A., Barth M., Schilling L., Fairlie W.D., Breit S.N., Unsicker K. (2001). Expression of growth differentiation factor-15/ macrophage inhibitory cytokine-1 (GDF-15/MIC-1) in the perinatal, adult, and injured rat brain. J. Comp. Neurol..

[6] Prus K., Sekuła M., Bilotta F. (2025). Biomarkers of acute brain injury. Curr. Opin. Anaesthesiol..

[7] Smith H.W., Marshall C.J. (2010). Regulation of cell signalling by uPAR. Nat. Rev. Mol. Cell Biol..

[8] Rasmussen L.J.H., Petersen J.E.V., Eugen-Olsen J. (2021). Soluble Urokinase Plasminogen Activator Receptor (suPAR) as a Biomarker of Systemic Chronic Inflammation. Front. Immunol..

[9] Chew-Harris J., Appleby S., Richards A.M., Troughton R.W., Pemberton C.J. (2019). Analytical, biochemical and clearance considerations of soluble urokinase plasminogen activator receptor (suPAR) in healthy individuals. Clin. Biochem..

[10] Hahm E., Wei C., Fernandez I., Li J., Tardi N.J., Tracy M., Wadhwani S., Cao Y., Peev V., Zloza A. (2017). Bone marrow-derived immature myeloid cells are a main source of circulating suPAR contributing to proteinuric kidney disease. Nat. Med..

[11] Enocsson H., Sjöwall C. (2015). Soluble urokinase plasminogen activator receptor—A valuable biomarker in systemic lupus erythematosus?. Clin. Chim. Acta.

[12] Sjöwall C., Martinsson K., Cardell K., Ekstedt M., Kechagias S. (2014). Soluble urokinase plasminogen activator receptor levels are associated with severity of fibrosis in non-alcoholic fatty liver disease. Transl. Res..

[13] Wlazel R.N., Szwabe K., Guligowska A., Kostka T. (2020). Soluble urokinase plasminogen activator receptor level in individuals of advanced age. Sci. Rep..

[14] Furman D., Campisi J., Verdin E., Carrera-Bastos P., Targ S., Franceschi C., Ferrucci L., Gilroy D.W., Fasano A., Miller G.W. (2019). Chronic inflammation in the etiology of disease across the life span. Nat. Med..

[15] Silva-Bermudez L.S., Klüter H., Kzhyshkowska J.G. (2024). Macrophages as a Source and Target of GDF-15. Int. J. Mol. Sci..

[16] Ratnam N.M., Peterson J.M., Talbert E.E., Ladner K.J., Rajasekera P.V., Schmidt C.R., Dillhoff M.E., Swanson B.J., Haverick E., Kladney R.D. (2017). NF-κB regulates GDF-15 to suppress macrophage surveillance during early tumor development. J. Clin. Investig..

[17] Talbot N.P., Lakhal S., Smith T.G., Privat C., Nickol A.H., Rivera-Ch M., León-Velarde F., Dorrington K.L., Mole D.R., Robbins P.A. (2012). Regulation of hepcidin expression at high altitude. Blood.

[18] Wiklund F.E., Bennet A.M., Magnusson P.K.E., Eriksson U.K., Lindmark F., Wu L., Yaghoutyfam N., Marquis C.P., Stattin P., Pedersen N.L. (2010). Macrophage inhibitory cytokine-1 (MIC-1/GDF15): A new marker of all-cause mortality. Aging Cell.

[19] Wallentin L., Lindhagen L., Ärnström E., Husted S., Janzon M., Johnsen S.P., Kontny F., Kempf T., Levin L., Lindahl B. (2016). Early invasive versus noninvasive treatment in patients with non-ST-elevation acute coronary syndrome (FRISC-II): 15 year follow-up of a prospective, randomised multicentre study. Lancet.

[20] Anand I.S., Kempf T., Rector T.S., Tapken H., Allhoff T., Jantzen F., Kuskowski M., Cohn J.N., Drexler H., Wollert K.C. (2010). Serial measurement of growthdifferentiation factor-15 in heart failure: Relation to disease severity and prognosis in the Valsartan Heart Failure Trial. Circulation.

[21] Andersson J., Fall T., Delicano R., Wennberg P., Jansson J.H. (2020). GDF-15 is associated with sudden cardiac death due to incident myocardial infarction. Resuscitation.

[22] Bonaca M.P., Morrow D.A., Braunwald E., Cannon C.P., Jiang S., Breher S., Sabatine M.S., Kempf T., Wallentin L., Wollert K.C. (2011). Growth differentation factor-15 and risk of recurrent events in patients stabilized after acute coronary syndrome. Observations from PROVE IT-TIMI 22. Arterioscler. Thromb. Vasc. Biol..

[23] Hijazi Z., Oldgren J., Lindbäck J., Alexander J.H., Connolly S.J., Eikelboom J.W., Ezekowitz M.D., Held C., Hylek E.M., Lopes R.D. (2016). The novel biomarker-based ABC (age, biomarkers, clinical history)-bleeding risk score for patients with atrial fibrillation: A derivation and validation study. Lancet.

[24] Cortés M., Lumpuy-Castillo J., García-Talavera C.S., Rivera M.B.A., de Miguel L., Bollas A.J., Romero-Otero J.M., Chapel J.A.E., Taibo-Urquía M., Pello A.M. (2025). New Biomarkers in the Prognostic Assessment of Acute Heart Failure with Reduced Ejection Fraction: Beyond Natriuretic Peptides. Int. J. Mol. Sci..

[25] Gadd D.A., Smith H.M., Mullin D., Chybowska O., Hillary R.F., Kimenai D.M., Bernabeu E., Cheng Y., Fawns-Ritchie C., Campbell A. (2024). DNAm scores for serum GDF15 and NT-proBNP levels associate with a range of traits affecting the body and brain. Clin. Epigenet..

[26] Meloux A., Rigal E., Rochette L., Cottin Y., Bejot Y., Vergely C. (2018). Ischemic Stroke Increases Heart Vulnerability to Ischemia-Reperfusion and Alters Myocardial Cardioprotective Pathways. Stroke.

[27] Lee J., Giannaris P.S., Yilmaz C.E., Yilmaz G. (2025). Emerging biomarkers in ischemic stroke. Vessel. Plus.

[28] Zhou Y., Dou L., Wang L., Chen J., Mao R., Zhu L., Liu D., Zheng K. (2025). Growth and differentiation factor 15: An emerging therapeutic target for brain diseases. Biosci. Trends.

[29] Ni W., Han Y., Zhao J., Cui J., Wang K., Wang R., Liu Y. (2016). Serum Soluble Urokinase Type Plasminogen Activator Receptor as a Biological Marker of Bacterial Infection in Adults: A Systematic Review and Meta-Analysis. Sci. Rep..

[30] Desmedt S., Desmedt V., Delanghe J.R., Speeckaert R., Speeckaert M.M. (2017). The Intriguing Role of Soluble Urokinase Receptor in Inflammatory Diseases. Crit. Rev. Clin. Lab. Sci..

[31] Marsland A.L. (2021). suPAR: A Newer Biomarker of Systemic Chronic Inflammation. Brain Behav. Immun..

[32] Sajanti A., Hellström S., Bennett C., Srinath A., Jhaveri A., Cao Y., Takala R., Frantzén J., Koskimäki F., Falter J. (2025). Soluble Urokinase-Type Plasminogen Activator Receptor and Inflammatory Biomarker Response with Prognostic Significance after Acute Neuronal Injury—A Prospective Cohort Study. Inflammation.

[33] Yamaguchi H., Nishiyama M., Tokumoto S., Ishida Y., Tomioka K., Aoki K., Seino Y., Toyoshima D., Takeda H., Kurosawa H. (2021). Elevated Cytokine, Chemokine, and Growth and differentiation Factor-15 Levels in Hemorrhagic Shock and Encephalopathy Syndrome: A Retrospective Observational Study. Cytokine.

[34] Yamaguchi H., Nishiyama M., Tomioka K., Hongo H., Tokumoto S., Ishida Y., Toyoshima D., Kurosawa H., Nozu K., Maruyama A. (2022). Growth and Differentiation Factor-15 as a Potential Prognostic Biomarker for Status-Epilepticus-Associated-With-Fever: A Pilot Study. Brain Dev..

[35] Kilinc Y.B., Kilinc E., Danis A., Hanci F., Turay S., Ozge A., Bolay H. (2023). Mitochondrial Metabolism Related Markers GDF-15, FGF-21, and HIF-1αAre Elevated in Pediatric Migraine Attacks. Headache.

[36] Méloux A., Dogon G., Rigal E., Rochette L., Bejot Y., Vergely C. (2024). Proximal and distant expression of growth differentiation factor 15 (GDF15) correlate with neurological deficit following experimental ischemic stroke. PLoS ONE.

[37] Wang J., Zhang T., Xu F., Gao W., Chen M., Zhu H., Xu J., Yin X., Pang J., Zhang S. (2023). GDF-15 at admission predicts cardiovascular death, heart failure, and bleeding outcomes in patients with CAD. ESC Heart Fail..

